# A Review of Plant Vacuoles: Formation, Located Proteins, and Functions

**DOI:** 10.3390/plants8090327

**Published:** 2019-09-05

**Authors:** Xiaona Tan, Kaixia Li, Zheng Wang, Keming Zhu, Xiaoli Tan, Jun Cao

**Affiliations:** Institute of Life Sciences, Jiangsu University, Zhenjiang 212013, China (X.T.) (K.L.) (Z.W.) (K.Z.)

**Keywords:** plant vacuole, lytic vacuole, protein storage vacuole, vacuole iron transporter

## Abstract

Vacuoles, cellular membrane-bound organelles, are the largest compartments of cells, occupying up to 90% of the volume of plant cells. Vacuoles are formed by the biosynthetic and endocytotic pathways. In plants, the vacuole is crucial for growth and development and has a variety of functions, including storage and transport, intracellular environmental stability, and response to injury. Depending on the cell type and growth conditions, the size of vacuoles is highly dynamic. Different types of cell vacuoles store different substances, such as alkaloids, protein enzymes, inorganic salts, sugars, etc., and play important roles in multiple signaling pathways. Here, we summarize vacuole formation, types, vacuole-located proteins, and functions.

## 1. Discovery History of the Vacuoles

The term “vacuole” was first proposed by the famous French biologist Félix Dujardin and was used to represent the blank space of protozoan contractile vesicles [1]. A similar blank space was also observed in the leaves and roots of plants. Thus, the term was also adopted by plant biologists. In the early stages of vacuole research, methods of microscopic observation and neutral-red staining indicated that the vacuole was an acidic environment surrounded by membranes. At the end of the 19th century, de Vries believed that vacuoles were formed by special plastid-like precursors, called tonoplasts [2]. The vacuole is an important part of many cells. Mature cells of all terrestrial plants, most fungi, and algae (except prokaryotic cells) have vacuoles. However, there are no vacuoles in animal cells and in the immature plant cells, as well as some highly mature plant cells (such as stone cells). In mature plant cells, vacuoles can account for 90% of the cell’s volume. For a long time, the biochemical analysis of this organelle was hindered by a lack of technology, and the majority of knowledge came from the study of yeast bubbles. Some biochemical characterization of vacuole components and amino acid transport experiments were performed in yeast. In contrast, the study of plant vacuoles was limited to the localization of some vacuole components. In the early 1980s, the application of the method of separating vacuolar and purified vacuolar vesicles made biochemical and electrophysiological studies of plant vacuolar transporters feasible [3].

## 2. Formation Processes of Vacuoles

The early stages of vacuolar research were limited to examining vacuolar morphology, as described above. In recent years, however, improvements in technology have brought the research of vacuoles to the molecular level, including metabolomics, proteomics, T-DNA insertion mutants, and heterologous complementation.

**Figure 1:**
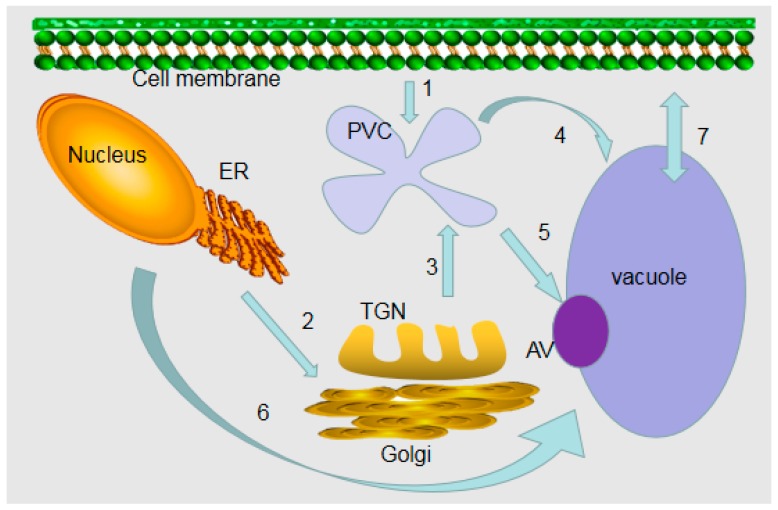
Model for the formation of plant vacuoles. (1) Endocytosis from the cell surface to a prevacuolar compartment (PVC). (2) Early secretory pathway from the endoplasmic reticulum (ER) to the late Golgi compartment. (3) Proteins are sorted into the PVC by an early biosynthetic vacuolar pathway. The Golgi apparatus/trans-Golgi network (TGN) system is important for biosynthetic traffic. (4) PVC is transferred to vacuoles via the late biosynthetic vacuole pathway. (5) PVC enters vacuoles through autophagic vacuoles (AV) by degradation or biosynthetic pathways. (6) Direct transport from ER to vacuole. (7) Transport of ions and solutes on vacuole membrane.

Experimental evidence indicates that the substances of the plant vacuole system come from the intracellular biosynthesis pathway and the endocytosis pathway. Biosynthesis pathways leading to vacuole formation include: (1) Early secretory pathway(from ER to late Golgi compartments): some proteins located in the vacuole are separated from the proteins transported to the cell surface; (2) Endocytosis of substances from the plasma membrane; (3) Autophagy; (4) Direct transmission of cytoplasm to vacuoles. Ultimately, specific proteins enter the vacuole and perform their functions through sorting and targeting mechanisms [4]. As in animal and yeast cells, the vacuolar transport pathway in plants begins with the endoplasmic reticulum (ER) (Figure 1). A conventional route of vacuole transport consists of a vesicle transport pathway including endoplasmic reticulum output mediated by vesicle and Golgi and post-Golgi transport [5,6]. For example, the tonoplast potassium channel AtTPK1 in *Arabidopsis* forms dimers, is controlled by ER quality control, and then reaches the vacuole through the Golgi apparatus [7]. An unconventional pathway of vacuolar transport is the direct transport from the ER to the vacuole without involving Golgi and Golgi-posterior vesicles [8,9]. Studies have shown that vacuole biogenesis and transport of plastid proteins and lipids can occur directly from the ER without Golgi involvement [10]. It is clear that these pathways are highly conserved in eukaryotes [11]. Recent studies have reviewed the commonalities and differences between different eukaryotic transport systems from the perspective of plants and have discussed plant vacuole substitution and trafficking pathways [9,12]. Proteins with N-terminal leading sequences or transmembrane domains are secreted by conventional protein secretion pathways. In contrast, proteins lacking signaling peptides are secreted by unconventional protein secretion pathways [8,13]. In addition, some studies have introduced two new glycosylated vacuolar GFP (green fluorescent protein) markers [14].

## 3. Different Types of the Vacuoles

Vacuoles account for most of the volume of mature cells in plants. Two types of vacuole, lytic vacuoles (LV) and protein storage vacuoles (PSV), appear sequentially during embryogenesis [15,16]. LVs contain hydrolases to degrade unwanted cellular substances, while PSVs accumulate large amounts of defense and storage proteins. Because of the degradation and storage functions exhibited by LVs and PSVs, respectively, some labeled proteins are used to distinguish them [15,17]. Recent studies have indicated that these two types of vacuoles share a transport protein [18,19]. They also noticed that in maturing seeds, the LVs were turned into PSVs, which was reversed during germination [20]. Most vacuolar soluble proteins are synthesized into larger precursors in the ER and then transported into vacuoles, in which become mature following the intervention of vacuolar processing enzymes (VPE) [21]. Most stored proteins are synthesized as precursors on ribosomes, processed by ER and Golgi apparatus, and transported to specialized vacuoles to perform their functions. This process usually requires proteolysis to promote stable storage [22,23]. PSVs have been shown to arise de novo in the cotyledons of the developing pea (*Pisum sativum*), and a similar mechanism may operate in *Medicago truncatula* embryos [24]. The results in *Arabidopsis* indicate that PSV arise by the remodeling of preexisting vacuoles rather than by de novo biogenesis of PSV [25]. In addition, the LVs play an important role in ion storage and homeostasis, cell degradation, stress buffering, and defense against pathogens. The rapid absorption or release of ions and water in the vacuole cavity enables plants to respond quickly and effectively to various environmental challenges [26].

## 4. Vacuole-Localized Proteins

Plant or animal cells contain up to 10,000 different types of proteins, while yeast cells contain about 5000. Each protein must be located in a precise intracellular compartment, cell membrane, or organelle to function properly [27]. Vacuolar-located proteins make up a very low amounts (1%) of total proteins [28]. Some transmembrane proteins and peripheral proteins are involved in vacuole activities, such as proton pumps, channel proteins, transport proteins, and solution carriers. Compared with some other organelles (such as mitochondria and chloroplasts), plant vacuoles are very fragile and difficult to isolate using traditional methods [29]. Large-scale isolation and identification of organelles, as well as qualitative and quantitative analysis of subcellular pathways, will contribute to a better understanding of the plant system.

Vacuoles were first isolated from protoplasts of young tomato root tip tissue by osmotic shock [30]. At present, there are two main strategies for the separation and enrichment of vacuolar proteins. One consists in preparing protoplasts, separating complete vacuoles from protoplasts, and then extracting vacuolar protein samples [31]. This technique is a key step in characterizing vacuolar transporters. It allows to study vacuoles from different plants and analyze their vacuole contents. Second, density gradients combined with isotope tagging can be used to distinguish membrane proteins from different organelles [32]. Besides, the patch clamp technology is commonly used to study the electrophysiological properties of vacuolar channels and transporters [33]. The first vacuole channel identified was the slow vacuole channel, which was later renamed TPC on the basis of its animal homolog. This approach showed that H^+^-ATPase and H^+^-PPase are localized on the same vacuole, and both pumps can acidify the vacuole cavity [34]. 

As an important model plant, *Arabidopsis* has been used for the study of biological functions. At present, there are reports on the isolation of vacuoles by osmotic shock of *Arabidopsis* leaves and cultured cell protoplasts [35,36]. In addition to *Arabidopsis*, other plant vacuoles have also been isolated, which will contribute to a better understanding of their functions (Table 1). In recent years, some intramembrane proteins have been characterized in the seed vacuoles [37]. Intracellular markers, called tonoplast intrinsic proteins (TIPs), have been used for vacuole biogenesis and identification [38].

## 5. Multifaceted Roles of Plant Vacuoles

Plant vacuoles are multifunctional organelles with differences not only in some basic properties but also in morphology and dynamics in different cell types and conditions [58]. Similar to the lysosomes of animal cells, they contain digestive hydrolases functioning in the degradation of extracellular and intracellular components [59,60]. In addition, vacuoles also transport a variety of secondary metabolites such as organic acids, glycosides and glutathione conjugates, alkaloids, and anthocyanin [58].

### 5.1. Vacuoles Can Be Used as Professional Repositories

Vacuoles are reservoirs of many metabolites, such as inorganic substances, organic acids, amino acids, and sugar in seeds and nutrient tissues [61,62]. Special cells in these organs accumulate proteins primarily as amino acid stores. The most common storage proteins are globulin, found in embryos, and glutenin, specific to cereal endosperm [63,64]. The results show that prolamins accumulation in the ER is an important step for the subsequent accumulation in storage vacuoles [63].

Many reports indicate that toxic pollutants in the environment have a serious negative impact on a variety of organisms [65]. Metals and metalloid pollutants come from natural or anthropogenic factors [66]. One study focused on the tolerance and accumulation mechanism of the heavy metals cadmium (Cd) and arsenic (As) and discussed how to use the knowledge collected on this subject to develop pollution-free crops and utilize phytoremediation [67]. Iron (Fe) is an essential micronutrient for both plant growth and human health. It can be used as a necessary cofactor for the electron transport chain of cell redox reactions involved in DNA biosynthesis, respiration, photosynthesis, and other reactions [68,69]. Iron deficiency leads to yellowing and growth retardation of young leaves, which leads to a decrease in photosynthetic efficiency and crop productivity [70]. Micronutrient malnutrition undermines the health and well-being of women and pre-school children in particular [71]. Conversely, excessive iron can cause severe dysfunction and cell damage, which is harmful to cells and organisms [72]. Metal contamination and toxicity in soil limits food production. Scientific research shows that biofortification is a way to solve hidden hunger by increasing the dietary iron content in staple food crops [73].

**Figure 2:**
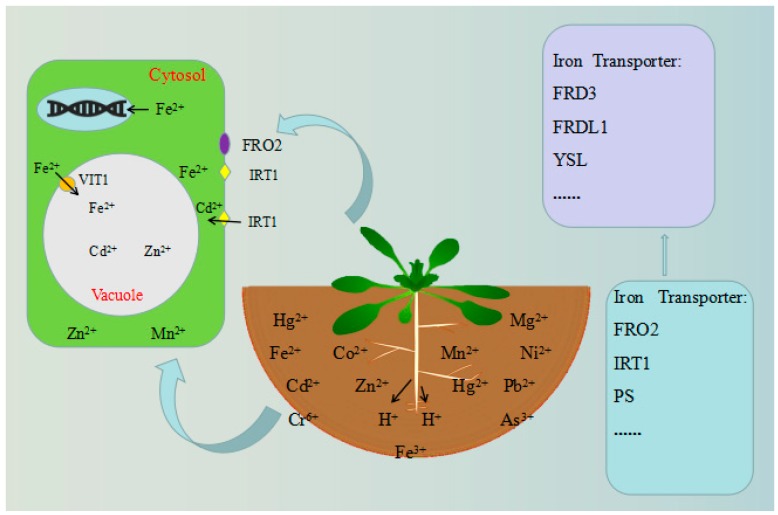
Tolerance mechanisms of model plants. Under the condition of iron deficiency, the four depicted mechanisms are upregulated in the root system. After that, iron is transported from the roots, through the xylem, to the shoots. Additionally, iron must also be transported to the cell compartments for utilization.

The storage of iron in seeds is a good example of plant vacuole storage of different heavy metals. At least 95% of the iron in *Arabidopsis* seeds is stored in vacuoles. However, in other seeds (such as *P. sativum*), the iron content of the vacuole is very low [74,75,76]. Iron is a rich element in most soils, but its solubility is low in aerobic environments, especially in alkaline calcareous soils [77]. Plants use two different strategies to absorb iron. In strategy I, plants, including dicots (such as cassava and *Arabidopsis*) and non-grass monocots, rely on the following processes: (1) plants secrete protons into the rhizosphere to reduce the pH of the soil, thereby increasing the solubility of ferric iron complexes (Fe^3+^); (2) the root protein ferric chelate reductase (FRO2) reduces Fe^3+^ into the more soluble Fe^2+^ (ferrous ion) on the root surface [78]; (3) the iron-regulated transporter 1 (IRT1)-type ferric transporter, of the zinc-iron transporter (ZIP) family, moves Fe^2+^ onto the cortical membrane of the root epidermal plasma membrane; (4) flavins are secreted to further facilitate the solubilization of ferric iron [79]. Under the condition of iron deficiency, the four mechanisms are upregulated in the root system. Incredibly, graminaceous plants have another unique strategy for absorbing iron, i.e. strategy II. This strategy has been described as a “chelation” strategy, similar to that used by many bacteria and fungi [80], and may result from adaptation to alkaline soils [81]. The plant secretes phytosiderophore (PS) into the rhizosphere to form the chelating complex Fe^3+^–PS, which is then absorbed into root cells by the yellow stripe 1 (YS1) transporter [82,83]. Iron is then transported from the roots through the xylem to the shoots, such as branches, leaves, and seeds, for use [69]. To reach its final destination, iron must also be transported to the appropriate cell compartments for use (Figure 2).

### 5.2. The Roles of Plant Vacuoles in Protein Degradation

Autophagy is the primary mechanism to control the degradation and the recycling of cellular components. As a degradation pathway, autophagy plays an important role in protein and organelle turnover [84]. It is involved in vacuolation and cell differentiation, which are essential for survival under stress conditions like nutritional deficiencies. On the other hand, autophagy is involved in some trafficking events [85]. Autophagy is also used to transport other types of cargo and vesicles produced by the ER, such as rubber and anthocyanins [86,87].

Studies have shown that the trans-Golgi network (TGN), provacuoles, and autophagosomes exhibit an acidic environment and contain lysosomal acid hydrolases [4]. Vacuoles usually contain high concentrations of phosphate, malate, citrate, aspartic acid, and glutamic acid [88]. The cytoplasm in autophagosomes degrades after being completely blocked. It is speculated that with the destruction of the inner boundary membrane, digestive enzymes are released. Digestive activity is restricted to the vacuole, which forms a tonoplast. After autophagy digestion is completed, autophagic vacuoles are incorporated into the central vacuole. However, when autophagic digestion is inhibited, autophagic vacuoles remain in the cytoplasm [4,89,90]. Studies have shown that the induction of autophagy is affected by the mitochondria and respiratory substrate supply, rather than by reducing the concentrations of sucrose and hexose phosphate [88]. The formation of autophagy vesicles is related to an increase in the intracellular proteolysis rate and decomposition of membrane polar lipids [91,92].

### 5.3. The Role of Vacuoles in Plant Metabolism

The vacuole is often enriched in plant specialized metabolites (PSMs), which contain more than 200,000 compounds [93]. Secondary metabolites were the first compounds found in vacuoles. Although most of the secondary compounds produced to prevent harmful environmental conditions, such as fighting pathogens and herbivores, are stable, they are converted into toxic compounds when cells are destroyed. For example, cyanoglucosinolates or glucosinolates are hydrolyzed to protect plants from their toxicity [50,94]. To some extent, these alterations are dependent on vacuolar transporters. In recent years, a detailed review of the vacuolar transport mechanisms of metabolites [93,95] and of the transport of flavonoids and alkaloids [95,96] has been carried out. Despite extensive research on glucosinolates, no vacuolar proteins have been found to transport these secondary compounds, and little is known about secondary compounds exported from vacuoles.

## 6. Our Prospect of Vacuole Research

Plant vacuoles are high dynamic and polymorphic, and their size depends on the cell type and growth conditions. Vacuolar fusion leads to increased longevity, and V-ATPase is an important regulator of pH homeostasis, multiple metabolic pathways, and longevity [97]. Many vacuolar transporters are transported from the ER through the Golgi apparatus [4]. This membrane transport can be accelerated during the closing of the stomata. When the stomata are opened, the number of vacuoles decreases, while their size increases. From a single confocal image slice, it was found that small bubbles fuse to form larger vacuoles when the stomata are opened [98]. Homogeneous membrane fusion steps are essential in vacuolar biosynthesis. Under some circumstances, two different types of vacuoles, protein storage vacuoles and lytic vacuoles, can also fuse to produce large central vacuoles [99]. Vacuoles distinguish different cellular components, such as proteins, sugars, tannins, organic acids, ions, and other secondary metabolites, and play a key role in plant response to different biological/abiotic signal transduction pathways. At present, many different intracellular transport pathways have been mapped and provide a structural framework for the existing concepts in vacuole physiology. Recent articles have also summarized two different approaches for vacuole defense against pathogens and discussed how plants use vacuole cell death to attack invading pathogens [100,101]. Recently, researchers have increased the research on the transport mechanisms of vacuolar transporters. It is also clear that plant cells have multiple mechanisms and transport pathways to sort vacuolar proteins. Nevertheless, there are still a lot of unresolved issues [102]. At present, our laboratory is studying the function of vacuole iron transporter in *Brassica napus L*. In addition, it is still difficult to detect the activity of vacuolar membranes because a certain degree of their enrichment in the corresponding cell fraction is required. Therefore, more work needs to be done to molecularly characterize vacuoles more accurately.

## Figures and Tables

**Table 1:** Partially localized proteins on vacuoles.

Classification	Name	Functions	References
Proton Pumps	Vacuolar-type H^+^-pumping ATP hydrolase (H^+^-ATPase, VHA)	For the acidification of the vacuole.	[39,40]
	H^+^-pumping pyrophosphatase (H^+^-PPase, AVP1)	For the acidification of vacuoles and the control of auxin transport.	[39,40,41]
Proton antiporters	Cation (Na^+^/K^+^) proton antiporters (NHXs)	To change the color of flowers.	[42]
	Na^+^/H^+^ antiporter (AtNHX1)	To mediate Na^+^ isolation in vacuoles and improve plant salt tolerance.	[43]
	Ca^2+^/H^+^ antiporters	To regulate plant processes, including ionic homeostasis and development.	[44]
	The characterization of the copper transporter COPT5	To export copper in vacuoles.	[45]
	Vacuolar anion exchanger AtCLCa, AtALMT9	Stomatal regulation and vacuole delivery of their anions.	[46,47]
ATP-binding cassette (ABC) transporters	MRPs, AtTAP2	For transporting glutathione conjugates and glucosidic acid conjugates.	[35,48]
Multidrug and toxic compound extrusion (MATE) transporters	SbMATE2	To transport secondary compounds such as alkaloids, cyano glucoside, and some flavonoids.	[49,50,51]
Heavy Metal Transporters	Vacuole iron transporter (VIT)	To regulate the synthesis of anthocyanins; resistance to heavy metal ions; to regulate cytosolic iron homeostasis.	[52,53]
	BnMEB2	Resistance to heavy metal ions	[54]
	Mn^2+^ transporters	Resistance to heavy metal ions	[55,56]
Vacuolar Sugar Transporters	AtSuc4	Resistance to heavy metal ions	[57]
